# Comparison of Supervised Classification Methods for Protein Profiling in Cancer Diagnosis

**Published:** 2007-07-19

**Authors:** Nadège Dossat, Alain Mangé, Jérôme Solassol, William Jacot, Ludovic Lhermitte, Thierry Maudelonde, Jean-Pierre Daurès, Nicolas Molinari

**Affiliations:** 1 IURC, Department of Biostatistic, Epidemiology and Clinical Research, Montpellier, France; 2 University of Montpellier I, Montpellier, France; 3 CHU Montpellier, Hôpital Arnaud de Villeneuve, Department of Cellular Biology, Montpellier, France; 4 CHU Montpellier, Hôpital Arnaud de Villeneuve, Department of Thoracic Oncology, Montpellier, France; 5 INSERM, U540, Montpellier, France; 6 Chu Nîmes, Hôspital Caremeau, Department of Medical Information, Nîmes, France

**Keywords:** mass spectrometry, Wilcoxon’s test, logistic regression, supervised classifications

## Abstract

A key challenge in clinical proteomics of cancer is the identification of biomarkers that could allow detection, diagnosis and prognosis of the diseases. Recent advances in mass spectrometry and proteomic instrumentations offer unique chance to rapidly identify these markers. These advances pose considerable challenges, similar to those created by microarray-based investigation, for the discovery of pattern of markers from high-dimensional data, specific to each pathologic state (e.g. normal vs cancer). We propose a three-step strategy to select important markers from high-dimensional mass spectrometry data using surface enhanced laser desorption/ionization (SELDI) technology. The first two steps are the selection of the most discriminating biomarkers with a construction of different classifiers. Finally, we compare and validate their performance and robustness using different supervised classification methods such as Support Vector Machine, Linear Discriminant Analysis, Quadratic Discriminant Analysis, Neural Networks, Classification Trees and Boosting Trees. We show that the proposed method is suitable for analysing high-throughput proteomics data and that the combination of logistic regression and Linear Discriminant Analysis outperform other methods tested.

## Introduction

Over recent years, scientific knowledge in cancer biology has progressed considerably. However, the practical impact of this research on screening, diagnosis, prognosis and monitoring remains limited. New methods must be developed to identify the physiological and pathological mechanisms in the origin and spread of tumors. Such approaches are essential for the discovery, identification and validation of new bio-markers. Recently, progress in mass spectrometry system, such as surface enhanced laser desorption/ionization time-of-flight (SELDI-TOF), has opened up interesting perspectives for identifying these markers or establishing specific protein profiles that may be used for cancer diagnosis (Adam, 2002; Petricoin, 2002; Zhang, 2004; Solassol, 2006). In this work, we considered SELDI raw data in attempt to discriminate cancer from benign diseases. After protein ionization and desorption with a laser, the mass spectrum is represented by the intensity of the proteins fixed on the chip (y-coordinate) as a function of the mass-to-charge (m/z) ratio (x-coordinate). From the spectra, the initial pre-processing steps are (a) the normalization and calibration to limit any bias caused by the instruments or the operator, (b) baseline subtraction, (c) peak detection, and (d) peak alignment to allow the same x-coordinate in all the spectra. One of the best challenges and the most important steps is then to reduce the high- dimension of these spectra to extract the discriminatory features or the best combination of markers capable of differentiating between two classes of interest (Duda, 2001). For this last step, spectra are processed using computerized algorithms based on multivariate statistical analyses. Several different mathematical algorithms have been applied to elucidate statistically significant differences such as cluster analysis, genetic algorithms, discriminate analysis, neural networks or hierarchical classification (Bauer, 1999; Petricoin, 2002; Vlahou, 2003; Wu, 2003).

In this work, we developed a three-step strategy to extract markers or combination of markers from high-dimensional SELDI data. From the pre-processing step, we detected 228 peaks, but among them some were not characteristic of the disease and were identically expressed in the two considered groups (cancer and benign disease). To allow an optimal identification of differentially expressed peaks, a preselection strategy of discriminating biomarkers combinations was chosen, rather than a simple filtering of the data by only a two-sided statistical test which was not taking into account the biomarkers inter-correlation. Next, we focused on different supervised classification methods due to the consideration that an *a priori* information coming from the training sample can allow the identification of the optimal diagnostic combinations. We compared the performance and the robustness of these various supervised classification methods and discussed their respective strengths and weaknesses.

## Data Set and Pre-Processing

The study involved a total of 170 serum samples collected at the Arnaud de Villeneuve University Hospital (Montpellier, France) with institutional approval: 147 patients with pathologically confirmed cancer and 23 patients suffering from a benign disease in the related organ. Whole blood was collected during fasting and all samples were processed within 1 h of collection and rapidly frozen at −80 °C before analysis. An anion-exchange fractionation procedure was performed before surface-enhanced laser desorption/ionisation time-of-flight mass spectrometry analysis. Serum samples were thus separated into six different pH gradient elution fractions, referred as to F1, F2, F3, F4, F5 and F6. Each fraction was randomly applied to a weak cation exchange ProteinChip array surface (CM10) in a 96-well format. F2 was not subjected to analysis due to the weak number of peaks detected in preliminary experiments. Arrays were read on a Protein Biological System II ProteinChip reader (Ciphergen Biosystem). Peak detection was performed using the ProteinChip Biomarker software (version 3.2.0, Ciphergen Biosystem Inc.). Spectra were background subtracted and the peak intensities were normalized to the total ion current of m/z between 2.5 and 50 kDa. Automatic peak detection was performed in the range of 2.5 to 50 kDa with the following settings: i) signal-to-noise ratio at 4 for the first pass and 2 for the second pass, ii) minimal peak threshold at 15% of all spectra, iii) cluster mass window at 0.5% of mass. The resulting CSV file containing absolute intensity and m/z ratio was exported into Microsoft Excel (Microsoft, Redmont) for subsequent analysis.

## Biomarkers Selection

Initially, a selection of the most discriminating biomarkers was carried out. The 228 peaks detected by Ciphergen software are aligned. A peak is defined as discriminating when the intensities of the individuals of the cancer group are significantly different than the reference group. Initially the peaks differentially expressed in the two groups were selected using the two-sided Wilcoxon’s test. After this preselection, a combination of discriminating peaks is required by using a logistic regression (Pepe, 2006).

### Wilcoxon’s test

The assumption that each peak intensity follows a normal distribution has been rejected using a Shapiro-Wilk normality test in each group. A two-sided Wilcoxon’s test was employed to test the *H*_0_ assumption of equality of the intensities in the two groups. We correct the loss of power induced by multiple tests by the false discovery rate (FDR) approach (Verhoeven, 2005; Benjamini, 1995). FDR is the expected proportion of type I errors among all significant results (*V*/*r*), where *V* is the number of type I errors (*“false discoveries”*), and *r* is the number of significant tests. A procedure to control FDR at level α was proposed by Benjamini and Hochberg (1995). That consists initially of ranking by ascending order the 228 p-values that we note now by : *p*_(1)_ ≤ *p*_(2)_ ≤ . . . ≤ *p*_(228)_, and *H*_(_*_i_*_)_ denote the null hypothesis corresponding to *p*_(_*_i_*_)_. The second stage consists of the search for *k* which is largest *i* for which:

$$p_{\left(i\right)}\leq \frac{\alpha }{228}i.$$

**Figure 1.**
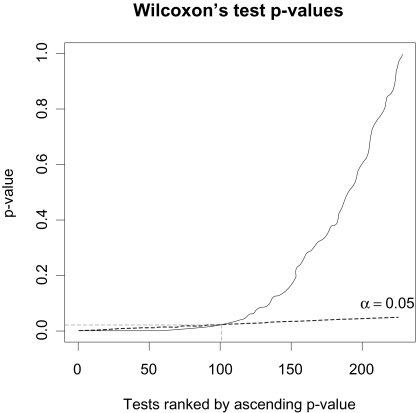
Application of Benjamini and Hochberg FDR control on the 228 Wilcoxon’s test p-values ranked by ascending order.

This resulting p-value *p*_(_*_k_*_)_ is the threshold p-value for each test taken individually, such as we reject all the null assumptions *H*_(1)_, …, *H*_(_*_k_*_)_ (Fig. 1). The null assumption has been rejected for *k =* 100 biomarkers.

### Binary logistic regression

The Wilcoxon’s retains the most discriminating peaks. On the other hand, the logistic regression combines several biomarkers to find the best model allowing classification in cancer/control groups. Let us consider the diagnosis variable *Y* to be modelled, which takes two values:

- *Y* = 1 for all the individuals that belong to the cancer group.
- *Y* = 0 for all the individuals that belong to the control group.

The outputs to be modelled *Y_i_|x_i_* follows a Bernouilli distribution of parameter π*_i_* *= P*(*Y_i_* *=* 1*|x_i_*), where *x_i_* is a vector line of the actual values for the explanatory variables. The *logit* of the multiple logistic regression (Hosmer, 2000) is given by

$$f(x)=logit\;(P(Y=1|x))=\beta_{0}+\beta_{1}x_{1}+\ldots +\beta_{p}x_{p}.$$

where (*x*_1_,…, *x_p_*) is a collection of *p* biomarkers selected in the model.

The classical model-building strategy is to find the most parsimonious model that explains the data. This provides a general and numerically stable model. To study the robustness of the logistic regression predictor’s selection, the two strategies of models-building forward and stepwise were employed. The significance level of the score chi-square for entering an effect into the model was fixed at 0.05 in the forward and stepwise logistic regressions. A significance level of 0.05 is considered in the Wald chi-square test to test if an effect must stay in the stepwise logistic regression which was implemented with SAS software (version 8.1). A weight of 1 and 147/23 was affected to the cancer and control groups, respectively. This weighting was employed because the control sample is subsampled.

The estimated logit in the forward selection is given by the following expression:

$$\begin{array}{l} \hat{\text{f}}(\text{x})=-13.46+0.58\times P_{3}^{F1}+0.34\times P_{22}^{F1}+29.76\\ \times P_{51}^{F3}+18.52\times P_{56}^{F3}-0.33\\ \times P_{136}^{F5}+6.37\times P_{156}^{F5} \end{array}$$

The estimated logit in the stepwise selection is given by the following expression:

$$\begin{array}{l} \hat{\text{f}}(\text{x})=-38.60-0.99\times P_{6}^{F1}+2.05\times P_{22}^{F1}+123.8\\ \times P_{51}^{F3}+61.86\times P_{56}^{F3}+1.88\times P_{129}^{F5}\\ -0.81\times P_{136}^{F5}+43.99\times P_{156}^{F5}-103.80\times P_{159}^{F5} \end{array}$$

where $P_{j}^{Fk}$ designed the *j*th peak in the original biomarkers matrix which contains 228 peaks and *Fk* indicates that this peak was detected in the *k*th fraction. The AUC for the two models were 0.988 for the forward strategy and 0.995 for the stepwise strategy. The forward and stepwise logistic regression modelled the logit with 6 and 8 biomarkers ranging from 3 to 48 kDa. The most stable peaks were the 5 common peaks of the two models i.e. $P_{22}^{F1}$, $P_{51}^{F3}$, $P_{56}^{F3}$, $P_{136}^{F5}$, $P_{156}^{F5}$. The model with these peaks is estimated by:

$$\begin{array}{l} \hat{\text{f}}(\text{x})=-11.85+0.41\times P_{22}^{F1}+25.55\times P_{51}^{F3}+20.66\\ \times P_{56}^{F3}-0.18\times P_{136}^{F5}+5.00\times P_{156}^{F5} \end{array}$$

for an AUC of 0.973.

These three models produced above were used in the comparison of the supervised classification techniques. For the moment, the AUC is the only criterion which makes it possible to evaluate the model in term of classification.

## Overview of the Supervised Classifications Used

Supervised classification techniques consist in a definition of a classification rule based on a training set for which the true class-label is known. The matrix *x* denotes the biomarkers matrix with *n* = 169 lines (i.e. *individuals*) and *p* columns, where *p* is the number of biomarkers retained in the different logistic regressions. Also, *x*_1_,…, *x_p_* denote the *p* biomarkers contained in the columns of *x*. The training data consist of *N* pairs {(*x*^(1)^, *y*^(1)^), (*x*^(2)^, *y*^(2)^), …, (*x*^(^*^N^*^)^, *y*^(^*^N^*^)^)} with *x*^(^*^i^*^)^ ∈ ℜ*^p^* and the *N* class-labels corresponding *y*^(i)^ ∈{ −1,1}. In the training set, the true class-label *y*^(^*^i^*^)^ adopted in the next sections are the following:

*y*^(^*^i^*^)^ = −1, if the sample point *x*^(^*^i^*^)^ belongs to the cancer group,
*y*^(^*^i^*^)^ = 1, if the sample point *x*^(^*^i^*^)^ belongs to the control group.

where, the *i*th line of the matrix *x*^(^*^i^*^)^ represents the *p* coordinates of the *i*th training sample point.

### Support vector machine

A Support Vector Machine (SVM) is a supervised learning technique that constructs an optimal separating hyperplane from the training set with an aim of classifying the test set (Vapnik, 1998; Hastie, 2001; Lee, 2004; Li, 2004). When the data are not linearly separable, one solution for the classification problem is to map the data into the feature space that is usually a higher-dimensional space using a function φ usually non linear. Thus, for *x*^(^*^i^*^)^ the *i*th vector in the original input space φ(*x*^(^*^i^*^)^) is the corresponding vector in the feature space. The value of the kernel function K on (*x*^(^*^i^*^)^, *x*^(^*^j^*^)^) computes the inner product of φ(*x*^(^*^i^*^)^) and φ(*x*^(^*^j^*^)^) in the feature space. The radial basis kernel is employed in this article. Its formulation is the following:

radial basis: K(*x*^(^*^i^*^)^, *x*^(^*^j^*^)^) = exp,(−‖*x*^(^*^i^*^)^–*x*^(^*^j^*^)^‖^2^*/c*),

where *c >* 0 is a scalar.

The search of the discriminant function *f* (*x*) = φ(*x*)*^T^* β + β_0_ is formulated into the following optimization problem

$$\underset{\beta ,\beta_{0}}{\text{min}}\frac{1}{2}{\left\|\beta \right\|}^{2}+\gamma \sum_{i-1}^{N}\xi_{i}$$

subject to ξ*_i_* ≥ 0,*y*^(^*^i^*^)^ (φ(x^(^*^i^*^)^)*^T^* β + β_0_) ≥ 1–ξ*_i_*,∀*_l_*, where γ > 0 is a constant and ξ*_i_* are the slack variables. This optimization problem is solved by maximizing the Lagrangian dual objective function. The solution β̂ for β has the form of linear combination of the terms *y*^(^*^i^*^)^ φ (*x*^(i)^) and β̂_0_ is the common value that solve *y*^(^*^i^*^)^[φ(*x*^(i)^)*^T^*β̂+ β̂_0_] = 1 for each *i*. The decision function can be written as

$$\hat{G}(x)=sign\left[\hat{f}(x)\right]=sign\left[\varphi {\left(x\right)}^{T}\hat{\beta }+{\hat{\beta }}_{0}\right].$$

In other words, if Ĝ(*x*^(^*^j^*^)^) = −1 then *j* th observation *x*^(^*^j^*^)^ of the sample test has a class-label *y*^(^*^j^*^)^ equal to −1 and will belong to the cancer group, else the class-label is equal to 1 and the individual *x*^(^*^j^*^)^ will belong to the control group.

### Linear discriminant analysis and quadratic discriminant analysis

Suppose that *f_k_* (*x*) is the density of the observations *x* in the *k* th class, and π*_k_* denote the prior probability of class *k*, where $\sum_{k=1}^{2}\pi_{k}=1$. The Bayes theorem gives us

$$P(Y=k|\;X=x)=\frac{f_{k}(x)\pi_{k}}{\sum_{l=1}^{2}f_{l}(x)\pi_{l}}\text{and}\;k\in \{1,2\}.$$

The classification rule for the test set is to affect the observation *x*′ at the *k* th class with maximal probability *P*(*Y = k*|*X* = *x*'). For linear and quadratic discriminant analysis, the densities *f_k_* are modelled as *p*-multivariate Gaussian (Webb, 2002; Lee, 2004). To compare the two classes *k* and *l*, the log-ratio was defined as

$$\text{log}\frac{P(Y=k|X=x)}{P(Y=l|X=x)}=\text{log}\frac{f_{k}(x)}{f_{l}(x)}+\text{log}\frac{\pi_{k}}{\pi_{l}}=\frac{\delta_{k}(x)}{\delta_{l}(x)}.$$

- Quadratic discriminant analysis (QDA) is the general discriminant problem, where the decision boundary {*x*: δ*_k_* (*x*) = δ*_l_* (x)} between the two classes is a quadratic equation in *x*. The quadratic discriminant function is defined as
  $$\begin{array}{l} \delta_{k}=-\frac{1}{2}\text{log}\left|\sum_{k}\right|-\frac{1}{2}{\left(x-\mu_{k}\right)}^{T}\sum_{k}^{-1}\left(x-\mu_{k}\right)\\ +\text{log}\pi_{k}. \end{array}$$
- Linear discriminant analysis (LDA) arises when the covariance matrix ∑*_k_* and ∑*_l_* are assume equals ∑*_k_* = ∑, ∀*k*. Then, the decision boundary between classes *k* and *l* is an equation linear in *x* in a *p*-dimensions hyperplane.

In practice, the true parameters of the Gaussian distributions are not known, but we can estimate them using the training set. Also, an estimate δ̂*_k_* of δ*_k_* can be obtained and the decision rule can be written as

$$\hat{G}(x)=\underset{k}{\text{arg}\text{max}{\hat{\delta }}_{k}(x)}$$

### Single-layer neural network

**Figure 2.**
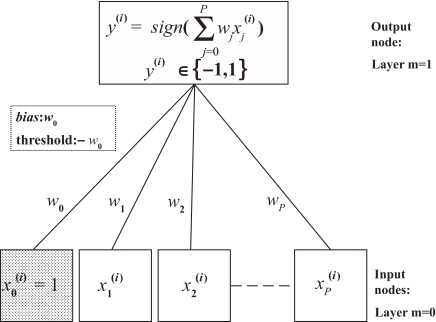
Schematic of a single-layer neural network.

Artificial neural networks (ANN) are learning algorithms that are modelled on the neural activity of the brain (Hastie, 2001; Dreyfus, 2002; Chen, 2004; Lee, 2004). Each node represents a neuron, and the connections represent the synapses (Fig. 2). A constant entry *x*_0_ = 1*_N_* is included in the whole perceptron entries, affected of a weight *w*_0_. The constant *w*_0_ is often referred as the bias and –*w*_0_ is called the threshold. Also, *x =* (*x*_0_, *x*_1_, *x*_2_, …, *x_p_*) denote the input variables such as *x_j_* ∈ ℜ*^N^*; *w =* (*w*_0_, *w*_1_, *w*_2_, …, *w_p_*) denote the associated weight vector. The training set is used to find the appropriate values of the synaptic weights vector (*w*_0_, *w*_1_, *w*_2_, …, *w_p_*) in neural networks to solve the classification problem. If the two classes are linearly separable, it exists a decision boundary {*x: w^T^* *x* = 0}. If *w^T^* *x >* 0, is in the first class and if *w^T^x >* 0, *x* is in the second class. A decision rule Ĝ(*x*) can be defined in terms of a linear function of the input *x* as follows

$$\hat{\text{G}}(x)=\text{sign}\;(w^{T}x),$$

where *sign*(*z*) denotes the sign of the quantity *z*. Let the risk function R(*w*) measures the success of a decision rule by comparing the true labels *y*^(^*^i^*^)^ with the predicted labels Ĝ(*x*^(^*^i^*^)^). The weight vector *w* is chosen to minimize the risk function. A current choice for the risk function is the sum of squared errors. The gradient descent procedure can be used to find optimum weights *ŵ* in term of risk, and the decision rule can be written as

$$\hat{\text{G}}(x)=\text{sign}\;(w^{T}x).$$

### Classification trees

**Figure 3.**
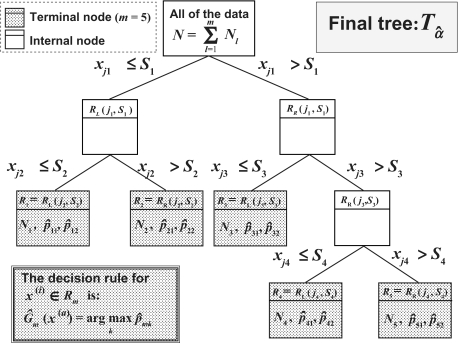
Schematic of an example of classification tree with 5 terminal nodes.

A classification tree is a multi-stage decision process that divides successively the whole of the *N* training sample observations in two homogeneous segments with regard to the class-labels by using the *p* biomarkers *x*_1_, …, *x_p_* (Hastie, 2001;Yang, 2005). The algorithm needs to select automatically a splitting rule for each internal node. This means determining a splitting variable $x_{j_{l}}{|}_{j_{l}\in \left\{1,2,\ldots ,p\right\}}$ with an associated threshold *S_l_* that has been used to partition the data set at each node in two regions : *R_L_*(*j_l_*, *s_l_*) = {*x|x_jl_* ≤ *s_l_*} and *R_R_*(*j_l_*, *s_l_*) = {*x|x_jl_* > *s_l_*}. For each splitting variable *x_jl_*, the threshold *s_l_* is determined by scanning through all of the inputs $x_{j_{l}}^{\left(i\right)}{|}_{i=1,2\ldots ,N}$, and the determination of the best pair (*j_l_*, *s_l_*) in term of maximization of the decrease in the node impurity function. In this article, the decrease in the node impurity function is expressed according to the Gini criterion. The splitting process is repeated on each of the two resulting regions of the previous step, and this until the stopping rule stops the process. The splitting process (Nakache, 2003) is stopped when the segment is pure (it contains subjects of the same class), if it contains identical observations, or if it contains a small number of subjects. Then this large tree is pruned using cost-complexity pruning. The final tree retained is noted by *T_â_* (Fig. 3). For the *K* class (here *K =* 2) and the *M* nodes in the final tree *T_â_*, the proportion of class *k* observations in terminal node *m* was computed as

$${\hat{p}}_{mk}=\frac{1}{N_{m}}\sum_{x^{\left(i\right)}\in \;R_{m}}1_{\left\{y^{\left(i\right)}=k\right\}}$$

where *N_m_*is the size of the training sample in the region *R_m_*. For *x* ∈ *R_m_* the decision rule is to affect *x* in the majority class in node *m*, and it can be written as

$${\hat{G}}_{m}(x)=\underset{k}{\text{arg}\text{max}\;{\hat{p}}_{mk}}$$

In other words, in the particular case where *K =* 2 for each final node *m* of the final tree the assignment rule can be also written in the following term:

If *p̂_mk_* ≥ 0 5 then the individuals of the node *m* are assigned to the class *k*, else they are assigned to the remaining class.

### Boosting trees

The purpose of boosting is to apply *M* times the weak classification algorithm on the weighted training data, so as to produce a sequence of weak classifiers G*_m_*(*x*), m = 1,2, …, *M* (Hastie, 2001; Fushiki, 2006). Then, a strong classifier is built by making a linear combination of the weighted sequence of weak classifiers. For a vector variables *X*, a classifier *G_m_*(X) produces a prediction of the class-label *Y* that belongs to {−1,1}. The error rate on the training sample is

$$\varepsilon_{m}=\frac{\frac{1}{N}\sum_{i=1}^{N}w_{m}^{\left(i\right)}I\left(y^{\left(i\right)}\neq G_{m}(x^{\left(i\right)})\right)}{\sum_{i=1}^{N}w_{m}^{\left(i\right)}}.$$

where $w_{m}^{\left(i\right)}$ is the weight associated to the *i* th observation of the training sample at the *m*th step. A weak classifier is one whose error rate is only slightly better than random guessing. The weights are initialized with $w_{1}^{\left(i\right)}=1/N{|}_{i=1;2,\ldots ,N}$. For each iteration *m =* 2,3,…, *M* the observation weights are modified and the classification algorithm is reapplied to the weighted observations. The error rate ε*_m_* is computed and the weights of the observations at the *m +* 1th step are recomputed as

$$\begin{array}{l} w_{m+1}^{\left(i\right)}=w_{m}^{\left(i\right)}.\text{exp}\left[\alpha_{m}.I\;(y^{\left(i\right)}\neq G_{m}(x^{\left(i\right)}))\right],\\ \;i=1,2\ldots ,N \end{array}$$

where α*_m_* *= log*((1–ε*_m_*)/ε*_m_*). In other words, at the step *m* the observations misclassified at the previous step have their weights increased (Nakache, 2003), and on the contrary the weights of the well classified observations are decreased. The predictions from all of them are then combined trough a weighted majority vote to produce the final prediction:

$$\hat{G}(x)=sign\left(\sum_{m=1}^{M}\alpha_{m}G_{m}(x)\right).$$

## Cross-validation

The cross-validation is applied on biomarker selections combined with different classification methods. The logistic regression, which took part at the preselection step, was also used as a discriminant analysis method in the cross-validation. The aim of this step is to validate our method of marker selections, while comparing the predictive power of the different supervised classification methods with this selection method. We applied the holdout method for the cross-validation. That consists on repeating the algorithm of decision rules construction described below, and to estimate their performances. First, the cross-validation consists on a random drawing of a training sample. The training sample size *N* was varied from 40%, 60%, and 80% of the total sample size *n* = 169. The remaining sample is named test sample. The features number of the training sample is limited to these *p* most discriminating features described above, such as *x* ∈ ℜ *_N×p_*. The decision rule *G*(*x*) is evaluated using the training set whose class-labels are known, and that for the different supervised classification techniques studied in this article. The class-label of each test sample observation is predicted using the decision rules *G*(*x*). The class-labels of the test sample being known, the predictions of the different methods can be evaluated by the calculation of TP, TN, FP, FN, where TP, TN, FP, FN means the number of true positive, true negative, false positive and false negative samples, respectively. These numbers are computed in each test set of the 1000 iterations of the cross-validation and summed. For each classification method, the sensitivity, the specificity and the accuracy was calculated to compare them. The sensitivity is defined as *TP/*(*TP+FN*) which represents the ability of a classification method to classify correctly the patients reached of cancer, and the specificity defined as *TN/*(*TN+FP*) the percentage of observation of the control sample correctly classified. The accuracy is defined as (*TP+TN*)/(*TP+TN+FP+FN*) and measures the percentage of whole of observations correctly classified. The cross-validation was applied to the three biomarkers selections using, under the R software, the package CaMassClass (www.r-project.org) dedicated to the treatment of Protein Mass Spectra (SELDI) Data.

## Results and Discussion

The goal of our article was to detect biomarkers and to assess their discriminating capacity using the different several supervised classification methods. In this way, we believe that cross-validation can answer to this question. We developed a three-step strategy to extract markers or combination of markers and to evaluate the robustness of these classifiers. First, protein peaks from 228 protein clusters were selected by a Wilcoxon test. Then, logistic regression models were used to construct two discriminating subsets of features composed of 6 and 8 protein peaks, ranging from 3 to 48 kDa, using forward (Table 1) and stepwise (Table 2) logistic regressions respectively. A third subset of discriminating markers (Table 3) was built by taking the intersection of the two first. Since there was no gold standard method for classification of mass spectrometry data, we were interested in comparing the performance and the robustness of different classification approaches. The unsupervised and supervised classification methods have been evaluated, but only the latter that showed the most satisfying results was presented in this paper.

The mean performance (accuracy, sensibility and specificity) of our classifiers on 1000 randomly generated 80:20, 60:40 and 40:60 set of samples were evaluated using different class-prediction models. The results showed that the forward logistic regression is better than the stepwise logistic regression in terms of accuracy and specificity. Interestingly, 5 protein peaks were common to the two models. A classifier with the protein peaks common to these two model selection methods allowed a more parsimonious model, as effective as the forward logistic regression (Table 3). Then it can be pointed out that the specificity was lower than the sensitivity and did not exceed the 0.86. The least effective supervised classification methods was the classification trees and the boosting trees that failed to correctly classify individuals of the control group. Although the sensitivity of both methods was acceptable, it was not the case for the specificity that was found lower than 0.36. Comparing to Quadratic Discriminant Analysis, the Linear Discriminant Analysis gave the best performance result to discriminate both samples achieving a mean classification accuracy of 0.93, a sensitivity of 0.95, and a specificity of 0.83 with a 80:20 cross-validation set samples (Table 1). The Linear Discriminant Analysis was slightly better than the Logistic Regression in terms of accuracy, and sensitivity. The results from SVM and Neural Networks were similar in terms of mean performance but showed a lower mean specificity (0.78) compared to Discriminant Analysis and Logistic Regression methods (0.86). Finally, the model selection robustness was confirmed by using different training sample sizes that varied from 40 to 80%. Interestingly, all the selection methods were stable with all the training sample size tested, except for Quadratic Discriminant Analysis. The Linear Discriminant Analysis remained the most robust method with a mean specificity ranging from 0.82 to 0.83, and sensitivity from 0.93 to 0.95 with the different sample sizes tested (Table 1).

We showed that Linear Discriminant Analysis, Quadratic Discriminant Analysis, Logistic Regression, Support Vector Machine and Neural Networks were the five most robust supervised classification methods in our study. The combination of the two-sided Wilcoxon’s test and the Logistic Regression for the markers pre-selection and the Linear Discriminant Analysis seemed to be the more effective in term of classification of samples in control and cancer groups. We observed that once the most discriminating markers are selected, the results of sensitivity, specificity and accuracy can be radically different from one method to another. The choice of these classification methods depends on the data, on the choice made for the pre-selection and on the problem that has to be solved. Also, it is essential to test several classification methods on the selected biomarkers. The question of the bias between the selection method and the Discriminant Analysis can arise. Accordingly we evaluated the whole method (i.e. the preselection stage combined with the Discriminant Analysis) in a 5-fold cross-validation (Ambroise, 2002). If we consider the preselection method with the logistic regression forward, we found an accuracy of 0.8763, a sensitivity of 0.9027, and a specificity of 0.7. The combination of this selection method and this classification method is robust. But these performances could be better if the difference between sizes of the two groups had not been so important. The relatively low specificity obtained with our data could be explained by this strong imbalance in the size of both sample groups, or by the choice of a control group with high-risk of developping cancer. This last condition could explain the very low specificity observed with the Classification Trees and the Boosting Trees classification methods, which uses thresholds. In conclusion, this biomarkers selection method should be employed on other studies, to validate its robustness. It also would be interesting to ensure a medium term follow-up of this control group population to allow the reappraisal of benign condition and rule out the possibility of infra clinical and radiological cancer development in this group of patient. In this case, it could allow a correct reallocation of the patient in the correct group and a more efficient re-evaluation of the different classification methods. Finally, the potential markers selected should be clearly identified and annotated using extra purification such as standard chromatography and/or electrophoresis and analysis by peptide mass fingerprint using more resolutive MS techniques or peptide sequencing via tandem MS analysis. This identification presents several interesting features, particularly during the discovery phase, by adding a supplementary validation phase using independent immunological methods, such as ELISA, and by increasing the predictive value of the molecular signature.

## Figures and Tables

**Table 1.** Cross-validation forward.

	**Training sample size (%)**	**TP**	**TN**	**FP**	**FN**	**Sensitivity**	**Specificity**	**Accuracy**
**Binary logistic regression**	0.4	82371	11148	2852	5629	0.9360	0.7963	0.9169
	0.6	53525	7547	1453	4475	0.9228	0.8386	0.9115
	0.8	26657	4289	711	2343	0.9192	0.8578	0.9102
**SVM**	0.4	83580	9666	4334	4420	0.9498	0.6904	0.9142
	0.6	55035	6761	2239	2965	0.9489	0.7512	0.9223
	0.8	27677	3868	1132	1323	0.9544	0.7736	0.9278
**LDA**	0.4	82465	11603	2397	5535	0.9371	0.8288	0.9222
	0.6	54676	7510	1490	3324	0.9427	0.8344	0.9281
	0.8	27483	4159	841	1517	0.9477	0.8318	0.9306
**QDA**	0.4	82687	6647	7353	5313	0.9396	0.4748	0.8758
	0.6	52177	6695	2305	5823	0.8996	0.7439	0.8787
	0.8	25860	4064	936	3140	0.8917	0.8128	0.8801
**Neural Networks**	0.4	86408	10132	4830	1592	0.9819	0.6772	0.9376
	0.6	56932	6819	2181	1068	0.9816	0.7577	0.9515
	0.8	28535	3828	1172	465	0.9840	0.7656	0.9519
**Classification Trees**	0.4	80945	3508	10492	7055	0.9198	0.2506	0.8280
	0.6	53202	2554	6446	4798	0.9173	0.2838	0.8322
	0.8	26804	1354	3646	2196	0.9243	0.2708	0.8282
**Boosting Trees**	0.4	84585	4921	9153	3415	0.9612	0.3497	0.8769
	0.6	55461	3278	5918	2539	0.9562	0.3565	0.8741
	0.8	27708	1668	4580	1292	0.9554	0.2670	0.8334

**Table 2.** Cross-validation stepwise.

	**Training sample size (%)**	**TP**	**TN**	**FP**	**FN**	**Sensitivity**	**Specificity**	**Accuracy**
**Binary logistic regression**	0.4	78024	9594	4406	9976	0.8866	0.6853	0.8590
	0.6	50115	6878	2122	7885	0.8641	0.7642	0.8506
	0.8	24871	3942	1058	4129	0.8576	0.7884	0.8474
**SVM**	0.4	81876	7211	6789	6124	0.9304	0.5151	0.8734
	0.6	53604	5122	3878	4396	0.9242	0.5691	0.8765
	0.8	26802	2941	2059	2198	0.9242	0.5882	0.8748
**LDA**	0.4	79379	10253	3747	8621	0.9020	0.7324	0.8787
	0.6	52929	6682	2318	5071	0.9126	0.7424	0.8897
	0.8	26691	3614	1386	2309	0.9204	0.7228	0.8913
**QDA**	0.4	83320	3909	10091	4680	0.9468	0.2792	0.8552
	0.6	52004	5052	3948	5996	0.8966	0.5613	0.8516
	0.8	25690	3219	1781	3310	0.8859	0.6438	0.8503
**Neural Networks**	0.4	85758	7750	6694	2242	0.9745	0.5366	0.9128
	0.6	56802	5317	3683	1198	0.9793	0.5908	0.9271
	0.8	28527	2891	2541	473	0.9837	0.5322	0.9125
**Classification Trees**	0.4	81399	3459	10541	6601	0.9250	0.2471	0.8319
	0.6	53351	2592	6408	4649	0.9198	0.2880	0.8350
	0.8	26680	1388	3612	2320	0.9200	0.2776	0.8255
**Boosting Trees**	0.4	80072	3654	7799	1401	0.9828	0.3190	0.9010
	0.6	52540	2480	5605	849	0.9841	0.3067	0.8950
	0.8	26102	1408	5322	455	0.9829	0.2092	0.8264

**Table 3.** Cross-validation for common peaks.

	**Training sample size (%)**	**TP**	**TN**	**FP**	**FN**	**Sensitivity**	**Specificity**	**Accuracy**
**Binary logistic regression**	0.4	80169	10970	3030	7831	0.9110	0.7836	0.8935
	0.6	52459	7542	1458	5541	0.9045	0.8380	0.8955
	0.8	26258	4256	744	2742	0.9054	0.8512	0.8975
**SVM**	0.4	81890	9144	4856	6110	0.9306	0.6531	0.8925
	0.6	54293	6543	2457	3707	0.9361	0.7270	0.9080
	0.8	27333	3769	1231	1667	0.9425	0.7538	0.9148
**LDA**	0.4	81219	11258	2742	6781	0.9229	0.8041	0.9066
	0.6	54271	7380	1620	3729	0.9357	0.8200	0.9202
	0.8	27320	4021	979	1680	0.9421	0.8042	0.9218
**QDA**	0.4	78654	8663	5337	9346	0.8938	0.6188	0.8560
	0.6	50593	6991	2009	7407	0.8723	0.7768	0.8595
	0.8	25080	4048	952	3920	0.8648	0.8096	0.8567
**Neura Networks**	0.4	86002	9173	5197	1998	0.9773	0.6383	0.9297
	0.6	56730	6180	2820	1270	0.9781	0.6867	0.9390
	0.8	28511	3341	1659	489	0.9831	0.6682	0.9368
**Classification Trees**	0.4	80836	3530	10470	7164	0.9186	0.2521	0.8271
	0.6	53349	2623	6377	4651	0.9198	0.2914	0.8354
	0.8	26795	1439	3561	2205	0.9240	0.2878	0.8304
**Boosting Trees**	0.4	79569	2546	9603	1164	0.9856	0.2096	0.8841
	0.6	52099	1478	8392	852	0.9839	0.1497	0.8529
	0.8	25924	708	9136	440	0.9833	0.0719	0.7355
